# Pathogenesis of *Streptococcus* urinary tract infection depends on bacterial strain and β-hemolysin/cytolysin that mediates cytotoxicity, cytokine synthesis, inflammation and virulence

**DOI:** 10.1038/srep29000

**Published:** 2016-07-07

**Authors:** Sophie Y. Leclercq, Matthew J. Sullivan, Deepak S. Ipe, Joshua P. Smith, Allan W. Cripps, Glen C. Ulett

**Affiliations:** 1 School of Medical Science, and Menzies Health Institute Queensland, Griffith University, Parklands 4222, Australia; 2 Research and Development Center, Ezequiel Dias Foundation (Funed), Belo Horizonte, MG, Brazil

## Abstract

*Streptococcus agalactiae* can cause urinary tract infection (UTI) including cystitis and asymptomatic bacteriuria (ABU). The early host-pathogen interactions that occur during *S. agalactiae* UTI and subsequent mechanisms of disease pathogenesis are poorly defined. Here, we define the early interactions between human bladder urothelial cells, monocyte-derived macrophages, and mouse bladder using uropathogenic *S. agalactiae* (UPSA) 807 and ABU-causing *S. agalactiae* (ABSA) 834 strains. UPSA 807 adhered, invaded and killed bladder urothelial cells more efficiently compared to ABSA 834 via mechanisms including low-level caspase-3 activation, and cytolysis, according to lactate dehydrogenase release measures and cell viability. Severe UPSA 807-induced cytotoxicity was mediated entirely by the bacterial β-hemolysin/cytolysin (β-H/C) because an β-H/C-deficient UPSA 807 isogenic mutant, UPSA 807Δ*cylE*, was not cytotoxic *in vitro*; the mutant was also significantly attenuated for colonization in the bladder *in vivo*. Analysis of infection-induced cytokines, including IL-8, IL-1β, IL-6 and TNF-α *in vitro* and *in vivo* revealed that cytokine and chemokine responses were dependent on expression of β-H/C that also elicited severe bladder neutrophilia. Thus, virulence of UPSA 807 encompasses adhesion to, invasion of and killing of bladder cells, pro-inflammatory cytokine/chemokine responses that elicit neutrophil infiltration, and β-H/C-mediated subversion of innate immune-mediated bacterial clearance from the bladder.

*Streptococcus agalactiae*, also known as Group B streptococcus, is a common commensal of the human gastrointestinal and genitourinary tracts^1^,^2^,^3^. In addition to causing a broad range of infectious diseases in neonates, pregnant women, the elderly and the immunocompromised, this organism is responsible for approximately 2–3% of all urinary tract infections (UTI)^4^,^5^. *S. agalactiae* can cause various forms of UTI including asymptomatic bacteriuria (ABU), cystitis and pyelonephritis^4^,^6^. Whereas ABU is defined by an absence of clinical signs or symptoms of UTI, acute UTI typically involves presentations including dysuria, increased urinary frequency and/or urgency, and/or hematuria^7^,^8^. The mean duration of urinary frequency and urgency in non-pregnant women is 3–4 days^5^. Uncomplicated cystitis usually resolves quickly^9^, however, this form of *S. agalactiae* UTI can be difficult to treat in the setting of complications or patient co-morbidities^10^. A recent review of the overall burden of *S. agalactiae* UTI underscores the importance of these infections as a major public-health concern, with approximately 160,000 cases in the United States annually^11^.

The pathogenic mechanisms that underlie acute UTI due to *S. agalactiae* are poorly defined. Surface-expressed protein adhesin molecules, immune-evasion factors and toxins of *S. agalactiae* are known to contribute to disease pathogenesis in other forms of infection caused by *S. agalactiae*, as reviewed elsewhere^12^,^13^,^14^. However, few studies have examined the role of virulence factors in *S. agalactiae* UTI. *S. agalactiae* can bind to human bladder urothelial cells *in vitro* and induce potent production of interleukin (IL)-1α in the murine bladder following *in vivo* colonization^15^,^16^,^17^. *S. agalactiae* is able to colonize the bladder urothelium in a gentamicin-protected manner *in vivo* suggesting that the bacteria invade host cells^18^. Kulkarni *et al*.^19^ also demonstrated that *S. agalactiae* colonizes bladder urothelial cells and that the β hemolysin/cytolysin (β-H/C) of the bacteria induces inflammation but is dispensable for bladder colonization^19^. The processes of *S. agalactiae* adhesion to urothelial cells and induction of host innate immune responses as early stages of pathogenesis^20^ may also be influenced by bacterial growth in urine that can underpin some of the dynamics of microbial colonization of the urinary tract^21^. For example, a recent study showed that some ABU-causing *S. agalactiae* (ABSA) strains can grow in urine, in contrast to some uropathogenic *S. agalactiae* (UPSA) that are unable to grow in urine^22^.

The clinical relevance of acute UTI and ABU due to *S. agalactiae* highlights the need to better elucidate the early host-pathogen interactions underlying these different forms of infection. Here, we reveal distinct early colonization events encompassing urothelial cells, monocyte-derived macrophages (MDMs), whole bladder tissue, innate cytokine responses, neutrophil infiltration and clinical strains of acute UTI- and ABU-causing *S. agalactiae* that provide new insight into the host-pathogen interactions underpinning these infections. Comparisons of *in vitro* colonization-adhesion and invasion, infection-induced cell death, and innate immune responses alongside *in vivo* infection assays in the mouse UTI model reveal differences in adhesion, invasion and cytotoxicity of UPSA and ABSA towards urothelial cells, MDMs and bladder tissue. We reveal acute bladder neutrophilia in mice following infection with UPSA but not ABSA and provide evidence that inflammation, cytotoxicity, and neutrophil infiltration is mediated by the bacterial β-H/C, which also promotes the survival of *S. agalactiae* in the bladder.

## Results

### UPSA 807 adheres to and invades human bladder urothelial cells more efficiently compared to ABSA 834

**Figure 1:**
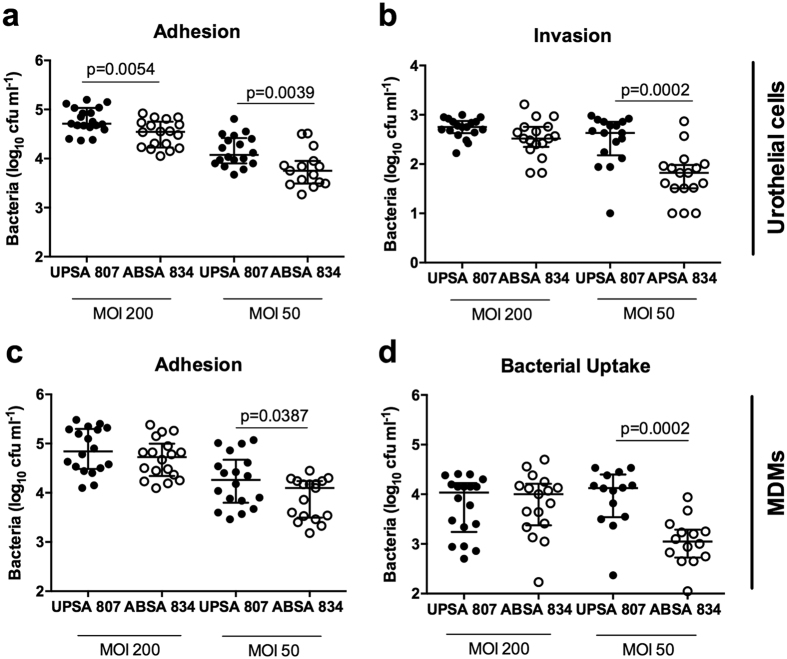
*In vitro* adherence and invasion of UPSA 807 and ABSA 834. UPSA and ABSA adherence at 1 h p.i (**a**) and invasion at 3 h p.i (**b**) were measured using human 5637 urothelial cells and gentamycin protection assays at MOIs of 200 and 50. The corresponding assays were performed using human U937 MDMs to assess adherence (**c**) and bacterial uptake (**d**) of UPSA and ABSA in macrophages. Data are pooled from four independent experiments each containing at least quadruplicate samples. The medians are shown with interquartile ranges and the data were compared (within MOI treatment groups) using Mann-Whitney U tests with P-values displayed.

We initially examined whether UPSA and ABSA might adhere to and invade urothelial cells with distinct efficiencies since these processes are critical early steps in UTI^20^. *In vitro* assays that were optimized to differentiate between adhered, intracellular and surviving *S. agalactiae*, showed that strain UPSA 807 adhered to and invaded human 5637 bladder urothelial cells at significantly higher levels compared to ABU strain ABSA 834 (Fig. 1a,b). Macrophages are also crucial innate defense cells in the bladder^23^,^24^,^25^, and interestingly we also observed significantly more adhesion to and uptake by human U937 MDMs by UPSA 807 compared to ABSA 834 (Fig. 1c,d). The differences observed were most significant at the lowest MOI tested (50), equating to 52.7% and 30.9% more UPSA 807 than ABSA 834 recovered at 1 h p.i. in urothelial cell and MDM adhesion assays, respectively. Invasion and bacterial uptake assays at 3 h p.i. demonstrated 84.5% and 91.6% more intracellular UPSA 807 than ABSA 834 in urothelial cells and MDMs (at MOI 50), respectively. Thus, UPSA 807 is significantly more adherent and invasive in human urothelial cells and MDMs compared to ABSA 834. Subsequent analysis of another clinical ABU strain, ABSA 1014, performed to compare with ABSA 834, showed similar patterns in adhesion compared to UPSA 807 (Supplementary Fig. S1a).

### UPSA 807 kills urothelial cells more effectively than ABSA 834 via a mechanism that involves low-level caspase-3 activation and cytolysis mediated by the β-H/C

**Figure 2:**
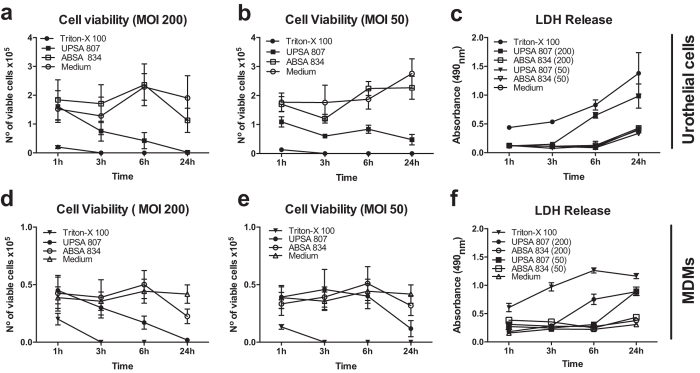
Cytotoxicity of UPSA 807 and ABSA 834 towards human 5637 urothelial cells and U937 MDMs. The levels of cytotoxicity for human urothelial cells are shown according to cell viability measures at MOI 200 (**a**), MOI 50 (**b**), and LDH release (**c**). Cell viability was determined using cell counts of trypan blue exclusion stained cells with LDH release assessed as a measured of cell lysis. Data for MDMs are shown for MOI 200 (**d**), MOI 50 (**e**), and LDH release (**f**). Cells treated with 2% Triton-X 100 (v/v) and RPMI media were used as positive and negative controls, respectively. Data are pooled from at least three independent assays each containing quadruplicate samples, and compared using AUC student’s t-test.

**Figure 3:**
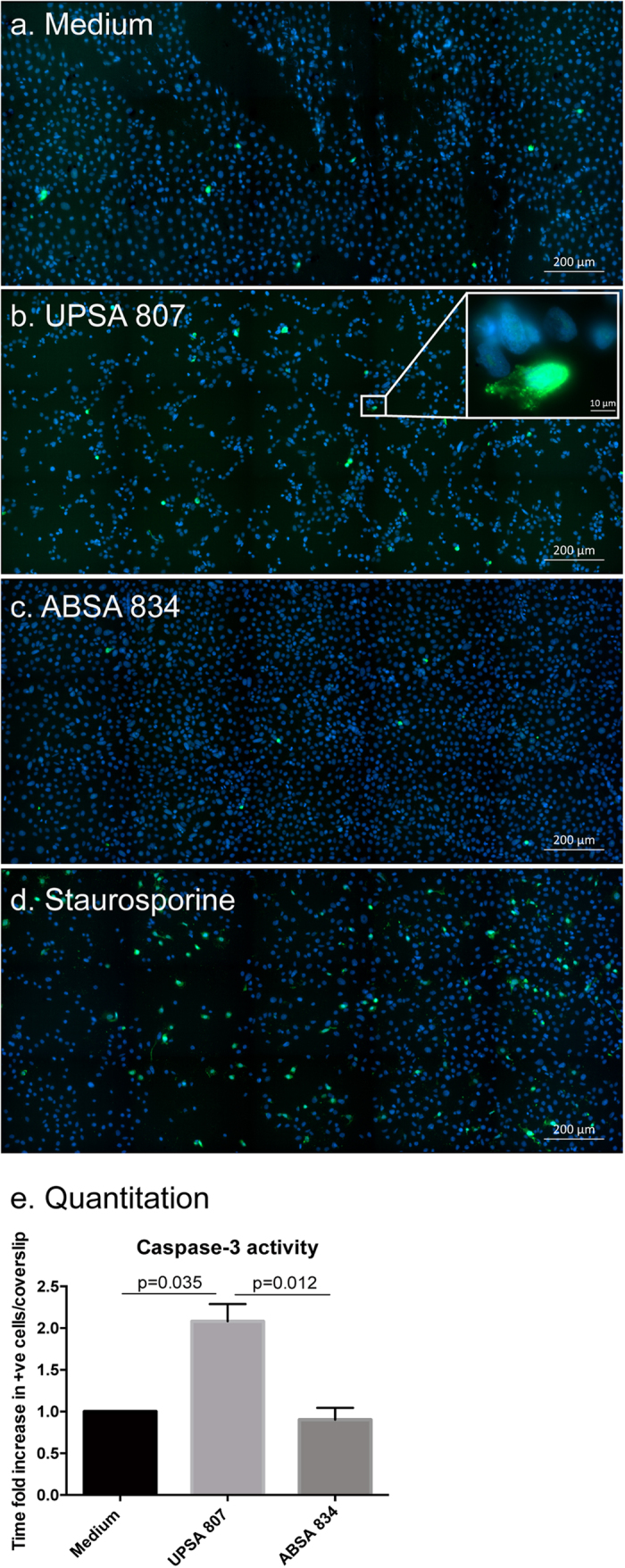
Activation of caspase-3 in urothelial cells exposed to UPSA 807 and ABSA 834. Caspase-3 activation was visualized in urothelial cells exposed to RPMI medium (**a**) (negative control), UPSA 807 (**b**) (MOI 200), ABSA 834 (**c**) (MOI 200) or staurosporine (**d**) (positive control). All images were captured 5 h p.i. at 20X magnification using tiling of twelve fields of view on a AxioImager.M2 microscope. Images show scale bars and are representative of three independent experiments. The levels of activation of caspase-3 were quantified (**e**) from three independent experiments each containing two coverslips and compared using one-way ANOVA with P-values displayed.

Infection-induced cell death can occur in urothelial cells during UTI^26^,^27^,^28^ and we next analyzed whether human bladder urothelial cells and MDMs might be killed following infection with UPSA 807 or ABSA 834. We observed severe cytotoxicity in urothelial cells and MDMs following infection with UPSA 807, but not ABSA 834, over the first 24 h p.i., according to cell viability measures and LDH release (Fig. 2). To investigate the mechanism by which UPSA 807 induces cellular cytotoxicity in bladder cells, we assessed caspase-3 activiation in infected cells, which revealed a statistically significant but modest level of caspase-3 activation following infection with UPSA 807 *in vitro;* the equivalent response of significant caspase-3 activation did not occur following infection with ABSA 834 (Fig. 3). Analysis of the activity of several caspases that lie upstream of caspase-3, using cell lysates from infected cultures at 5 h p.i., demonstrated no significant caspase-1, -8 or -9 activity following UPSA 807 infection (data not shown).

**Figure 4:**
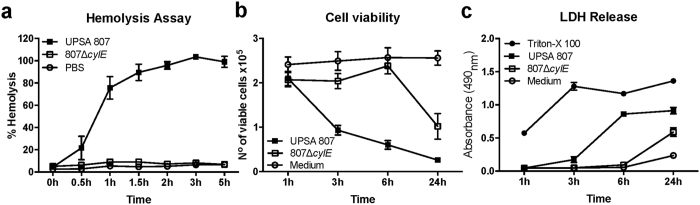
Comparative hemolytic and cytotoxic activities of UPSA 807 and its β-H/C-deficient isogenic mutant. Hemolytic activities of the bacteria towards erythrocytes in (**a**), are shown with measures of cell viability (**b**) and LDH release (**c**) for urothelial cells challenged with wt UPSA 807 and UPSA 807Δ*cylE* strains. Erythrocytes and urothelial cells exposed to 2% Triton-X-100 or RPMI medium were used as positive and negative controls, respectively. Data are pooled from three independent assays each containing quadruplicate samples and compared using AUC student’s t-test.

*S. agalactiae* cytotoxicity is often attributed to the bacterial β-H/C^29^ and we measured the hemolytic activity of UPSA 807 and ABSA 834 using an erythrocyte lysis assay. UPSA 807 exhibited significantly higher hemolytic activity compared to ABSA 834 (Supplementary Fig. S2a). To determine the function of the bacterial β-H/C in terms of urothelial cell death we generated an isogenic β-H/C-deficient UPSA 807 mutant (UPSA 807Δ*cylE*) and analyzed cytotoxicity. In contrast to wild-type (wt) UPSA 807, the 807Δ*cylE* mutant was not hemolytic (Fig. 4a) and, notably, was significantly less cytotoxic to urothelial cells, according to cell viability counts (Fig. 4b) and LDH release measures (Fig. 4c). Interestingly, UPSA 807 also exhibited significantly more rapid cytotoxicity towards urothelial cell viability compared to ABSA 1014, according to total cell counts and LDH release measures (Supplementary Fig. S1b,c) even though these two strains showed equivalent levels of hemolytic activity (Supplementary Fig. S1d).

### UPSA 807 induces a more rapid and robust pro-inflammatory cytokine response in human urothelial cells and MDMs compared to ABSA 834 *in vitro*

**Figure 5:**
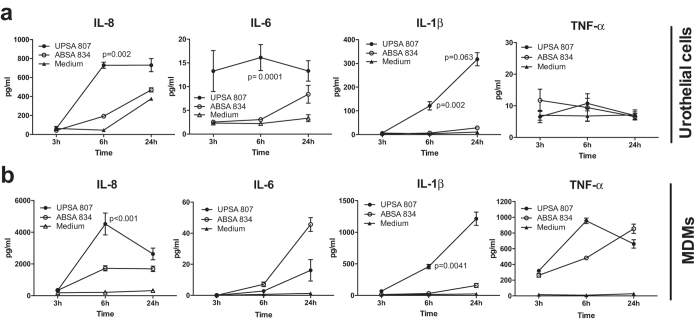
Cytokine responses induced in urothelial cells and MDMs exposed to *S. agalactiae in vitro*. Human 5637 urothelial cells (**a**) and human U937 MDMs (**b**) were challenged with UPSA 807, ABSA 834 or PBS as control (Medium). The concentrations of cytokines in supernatants collected at 3 h, 6 h and 24 h p.i. were quantified using ELISAs. Data were pooled from at least three independent experiments each containing quadruplicate samples, and compared using Kruskal-Wallis ANOVA and subsequent independent samples t-tests (or Mann-Whitney U tests) for pair-wise comparisons of UPSA 807 and ABSA 834 within time points with P-values displayed.

Analysis of the pro-inflammatory effects of UPSA 807 and ABSA 834 towards bladder urothelial cells showed that UPSA 807 induced significantly more IL-8, IL-1β and IL-6, but not TNF-α, compared to ABSA 834 (Fig. 5a). Time-course analysis (3 h, 6 h, and 24 h p.i.) showed significant production of several cytokines at 6 h p.i. in response to UPSA 807, in contrast to either insignificant responses or delayed production (24 h p.i.) following infection with ABSA 834. Similarly, MDMs infected with UPSA 807 produced significantly higher levels of IL-8 and IL-1β as early as 6 h p.i. and also produced higher levels of TNF-α compared to MDMs infected with ABSA 834 (Fig. 5b). In co-cultures of urothelial cells and MDMs that have been shown to synergize for synthesis of certain cytokines in response to uropathogenic *E. coli*^30^,^31^ we detected high levels of IL-8, IL-1β, IL-6 and TNF-α following UPSA 807 infection but the responses were not synergistic (data not shown). Thus, UPSA 807 induces a stronger and more rapid pro-inflammatory response than ABSA 834 in two cell types relevant to the bladder.

### UPSA 807 colonizes the bladder more rapidly than ABSA 834 but fails to persist

**Figure 6:**
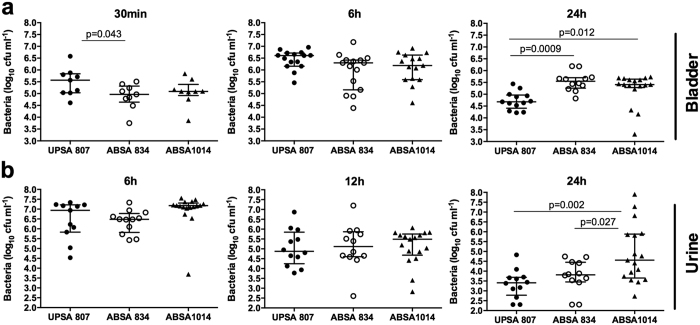
Bladder colonization in the murine model of UTI using UPSA 807 and ABSA strains 834 and 1014. C57BL/6 mice were challenged transurethrally and bacterial loads were determined using homogenized bladder tissues (30 min, 6 h and 24 h p.i.) and urine (6 h, 12 h and 24 h p.i.) collected at each timepoint. Data are pooled from two independent experiments (each containing 5–10 mice) and compared using Kruskal-Wallis ANOVA with Tukey’s multiple comparison test with P-values displayed.

In *ex vivo* adhesion assays, used to assess early adherence of bacteria to the bladder, significantly higher numbers of UPSA 807 were recovered from whole bladder at 0.5 h p.i. compared to ABSA 834 (Fig. 6a) (P = 0.043). However, there were no significant differences between the recoveries of these strains from the bladders of mice at 6 h p.i. following *in vivo* challenge (Fig. 6a). At 24 h p.i., in contrast, significantly fewer UPSA 807 were recovered from the bladders of mice compared to ABSA 834 (Fig. 6a) (P = 0.0009). Similarly higher recovery of ABSA 1014 compared to UPSA 807 was also observed at 24 h p.i. (Fig. 6a). There were no significant differences in bacterial loads in urine between any of the strains during the first 12 h p.i.; however, ABSA 1014 was recovered in significantly higher numbers compared to both UPSA 807 and ABSA 834 at 24 h p.i. (Fig. 6b), and while the average bacteriuria load was higher for ABSA 834 than UPSA 807 at 24 h p.i., this difference was not statistically significant (*P* = 0.052). Collectively, these data show that UPSA 807 adheres to the bladder more rapidly/efficiently compared to ABSA 834 but is also more rapidly cleared *in vivo*.

### UPSA 807 induces a more rapid cytokine/chemokine response in the bladder and a massive neutrophil infiltrate compared to the innate host response to ABSA 834

**Figure 7:**
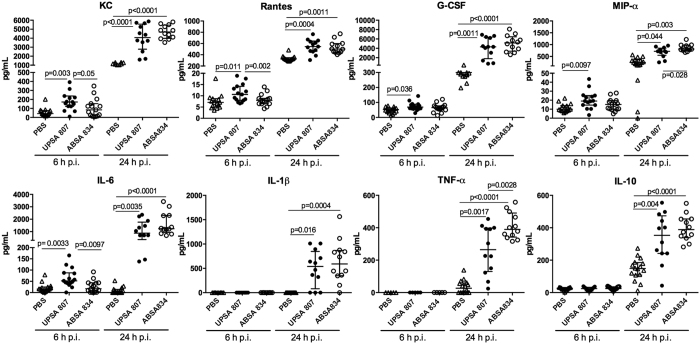
Cytokine production in the bladder *in vivo* following challenge of mice with UPSA 807 and ABSA 834. Cytokines were quantified in tissue homogenates using multiplex assays at 6 h and 24 h p.i. and compared to mice that received PBS. Data are pooled from two independent experiments and compared using Kruskal-Wallis ANOVA with Tukey’s multiple comparison test with P-values displayed.

**Figure 8:**
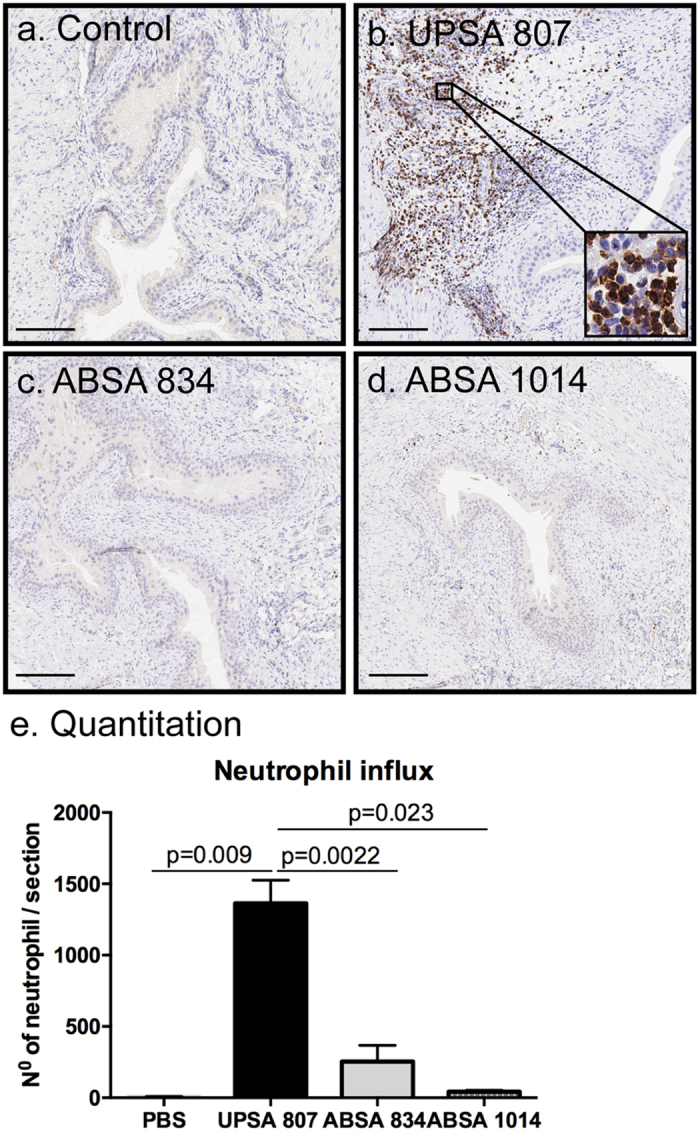
Histological analysis of neutrophil infiltrates in the bladder of mice induced by infection with UPSA 807, ABSA 834 or ABSA 1014. Representative tissue sections are shown for C57BL/6 mice that received PBS (**a**) Control), UPSA 807 (**b**), ABSA 834 (**c**) or ABSA 1014 (**d**). The tissue sections were prepared using bladders collected at 24 h p.i. and stained with anti-Ly6G antibody to show neutrophils (brown) followed by a light counterstain using Haematoxylin. Scale bars are 400 μm. Neutrophil infiltrates were quantified for entire sections of whole bladder (**e**) and are shown as mean ± SEM for triplicate tissue sections prepared from bladders that were collected from separate mice in independent experiments. Data were compared using independent samples t-test with P-values displayed.

We examined the innate response to UPSA 807 in the bladder to gain insight into the mechanisms underlying the more efficient clearance of UPSA 807 compared to ABSA. Several cytokines and chemokines were detected as earlier as 6 h p.i. in response to UPSA 807, including KC, Rantes, G-CSF, MIP-1α and IL-6 at levels significantly higher than the PBS group (Fig. 7). UPSA 807 induced KC, Rantes and IL-6 at significantly higher levels than ABSA 834 at 6 h p.i. but we did not detect any significant IL-1β, IL-10 or TNF-α responses at 6 h p.i. (Fig. 7). Interestingly, the levels of MIP-1α and TNF-α in mice infected with UPSA 807 were significantly lower than those for ABSA 834 at 24 h p.i.; and both strains induced high levels of all the other cytokines tested (Fig. 7). There were no significant differences in any of these cytokine profiles comparing mice challenged with UPSA 807 versus ABSA 1014 (Supplementary Fig. S3). In view of the rapid, significant differences in production of KC and Rantes *in vivo*, and given that IL-6 can act in the fashion of a classical chemokine^32^, we assessed bladder neutrophil infiltrates following infection. There were no significant differences in the tissue infiltrate between the groups at 6 h p.i. (data not shown). However, at 24 h p.i., mice infected with UPSA 807 exhibited massive neutrophil infiltration as evidenced by 177-fold more neutrophils compared to PBS control mice (Fig. 8a,b) and 5.3-fold more tissue neutrophils compared to mice infected with ABSA 834 (Fig. 8b,c) (P = 0.0022). A similarly minimal neutrophil infiltrate was observed for mice infected with ABSA 1014.

### UPSA 807-induced inflammation and local neutrophil infiltration in the bladder is mediated by β-H/C, which, paradoxically, contributes to bacterial survival *in vivo*

**Figure 9:**
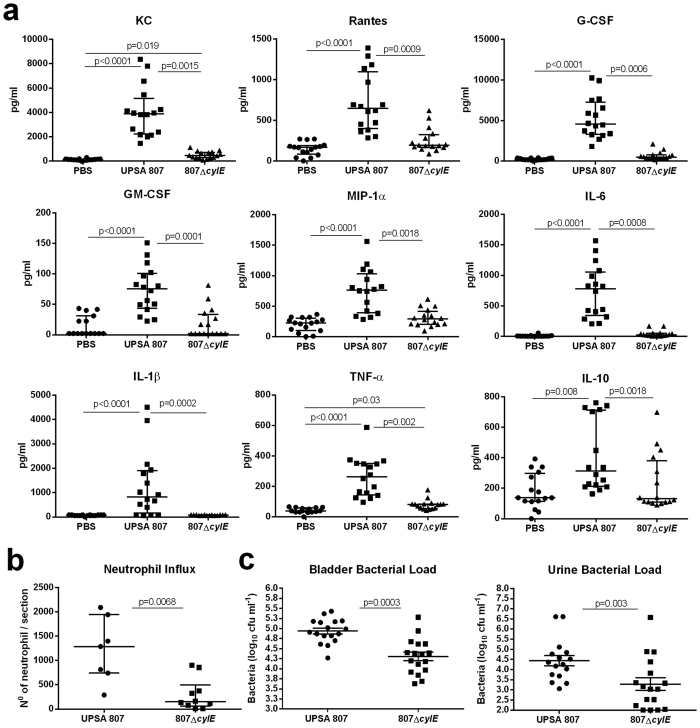
Innate immune responses and bacterial colonization in the bladders of mice infected with wt UPSA 807 and a β-H/C-deficient 807Δ*cylE* mutant. Cytokine levels in the bladder (**a**), neutrophil infiltrate (**b**) and bacterial loads in the bladder and urine (**c**) are shown for C57BL/6 mice challenged with UPSA 807, a β-H/C-deficient 807Δ*cylE* mutant or PBS at 24 h p.i. Data are pooled from two independent experiments (each containing 5–10 mice) and were compared using Kruskal-Wallis ANOVA (**a**), Mann-Whitney U test (**b**), or independent samples t-test (**c**) with P-values displayed.

We tested whether *S. agalactiae* β-H/C, which is known to induce cytokine/chemokine synthesis in other models of disease^33^,^34^ might be responsible for the observed effects induced by UPSA 807 in the bladder. Overall, cytokine production in the bladder was significantly lower in mice following infection with the β-H/C-deficient 807Δ*cylE* mutant compared with wt UPSA 807; only KC and TNF-α were significantly elevated above control levels detected in the PBS group (Fig. 9a). The observation of a broad absence of cytokine and chemokine responses in the bladders in mice infected with 807Δ*cylE* was consistent with a 5-fold lower neutrophil infiltrate in the bladder in mice infected with the 807Δ*cylE* mutant compared to wt UPSA 807 (Fig. 9b). Surprisingly, however, significantly fewer 807Δ*cylE* mutant were recovered from the bladder and urine at 24 h p.i. compared with wt UPSA 807 (Fig. 9c). Comparing the median numbers of bacteria recovered from the bladders showed 4.94 ± 0.07 wt UPSA 807 versus 4.31 ± 0.11 807Δ*cylE* mutant (*P* < 0.0001). Together, these data establish a key role for the bacterial β-H/C in eliciting inflammatory responses in the bladder including cytokine/chemokine synthesis and neutrophil infiltration, and in virulence by promoting the survival of *S. agalactiae* in the bladder.

## Discussion

The mechanisms of pathogenesis, including the virulence factors and early steps in bladder colonization that influence the progression of *S. agalactiae* UTI, are not well defined. We have defined the capacity of previously characterized *S. agalactiae* strains, isolated from cases of acute cystitis and ABU^4^,^6^,^22^, to adhere to, invade and activate human bladder cells and MDMs *in vitro*, and colonize and trigger innate immune responses in the mouse bladder *in vivo*. A major finding of this study is that UPSA 807 is more adhesive to and invasive in bladder urothelial cells and MDMs compared to ABSA 834; proficient adherence of UPSA 807 to human cells parallels infection in mice where we observed UPSA 807 colonizes the bladder more efficiently than ABSA 834 as early as 30 min post-infection. Strikingly, we found that proficient adherence of UPSA 807 to bladder cells leads to massive cytotoxicity via mechanisms that involve low-level, but significant, caspase-3 activation and cytolysis mediated almost entirely by the bacterial β-H/C. In addition, this study shows that the degree of early cytokine responses in the bladder, especially for IL-8 (KC in mice), IL-1β, IL-6 and Rantes, are defined by the infecting strain; whereas UPSA 807 triggers rapid responses associated with acute bladder neutrophilia ABSA 834 fails to elicit these responses. Finally, we establish a key role for the β-H/C in inducing early chemokine responses and bladder neutrophilia, and also, paradoxically, the ability of UPSA 807 to survive in the bladder *in vivo*.

The finding of proficient adherence of UPSA 807 to urothelial cells and bladder tissue is in agreement with prior observations on the adherence capacity of UPSA to bladder cells and tissue^15^,^16^. Notably, however, neither of these prior studies analyzed ABSA strains, cytotoxicity towards host cells, innate immune responses of chemokine activity and neutrophil infiltration, nor the role of the β-H/C in pathogenesis. The processes of adhesion to, invasion of and killing of host cells are well-described virulence strategies in the establishment of *E. coli* UTI^35^,^36^. Thus, the results of the current study suggest that these processes are important in shaping the progression of *S. agalactiae* UTI as well. It is important, however, to interpret the *in vitro* data, that show a proficiency of UPSA 807 for these virulence traits, in view of the limitations of the model used; these include restricted analysis of time points and infection ratios, a potential for cytotoxicity-induced antibiotic exposure to affect bacterial counts and inability to replicate complex processes that underpin *in vivo* host-pathogen interactions, as reviewed elsewhere^31^. Notwithstanding such limitations it is notable that the virulence characteristics of UPSA 807 in terms of the early interactions with host cells are not limited to bladder urothelial cells; our analysis of MDMs demonstrates that UPSA 807 also efficiently adheres to, is ingested by, and kills human macrophages. These cells are emerging as critical mediators of innate immune defense during UTI and also appear to modify effector adaptive immunity in the bladder^23^,^24^,^37^,^38^. Future studies aimed at defining the role of macrophages in *S. agalactiae* infection in the bladder would thus be of interest.

Early cytokine responses are crucial first steps in the host response to infection that is aimed at eradicating bacteria from the bladder. The finding that UPSA 807 induces a more rapid and stronger pro-inflammatory response comprising IL-8, IL-1β and IL-6 in urothelial cells *in vitro* compared to ABSA 834 within 6 h p.i. was comparable to the responses observed *in vivo* that comprised KC and IL-6 but not IL-1β. These differences in IL-1β and other cytokine production observed at 6 h p.i. probably reflect limitations in the *in vitro* model^31^, or species differences between human and mouse. However, broadly equivalent cytokine responses in urothelial cells and bladder induced by UPSA 807 and ABSA strains at 24 h p.i. provide evidence that the innate immune system has a considerable capacity to detect and respond to ABSA with multiple cytokine responses, even if the responses are slower compared to those induced by UPSA 807. The stronger, more rapid pro-inflammatory responses observed for UPSA 807 infection support the emerging concept that pro-inflammatory responses can drive some UTI symptoms in mice and humans, as reviewed elsewhere^20^. Heightened inflammatory responses, as a consequence of high bacterial burdens, have also been proposed to contribute to symptoms during *S. agalactiae* infection of joints^39^. The findings of the current study are also of interest in the context of recent clinical findings that show that ABU *E. coli* elicits the synthesis of several innate immune mediators, and the degree of these innate responses are influenced by host genetics^40^. The molecular basis of exaggerated host responses that drive disease versus attenuated responses that favor host defense have been shown to lie in promoter polymorphisms and transcription factor variants, as reviewed elsewhere^41^. Thus, future studies of the impact of host genetic background towards the degree of innate immune response to *S. agalactiae* UTI, and the factors of UPSA and ABSA that trigger these host responses, will be of interest.

We reasoned that divergent interactions between UPSA 807 and ABSA 834 and host cells are likely to be influenced by the distinctly different hemolytic activities of these strains, as well as potentially, differences in other factors such as capsular serotype, genetic sequence type, and different virulence gene repertoires. In this regard, we used an isogenic β-H/C-deficient mutant of UPSA 807 to determine the role of this known virulence factor in cytotoxicity, chemokine responses, bacterial fitness, and cell infiltration in the bladder during UTI. The finding of a major role of β-H/C in UPSA-driven cytotoxicity towards urothelial cells is consistent with findings in other disease models for *S. agalactiae*; for example, the β-H/C contributes to virulence mechanisms including cytotoxicity in dendritic cells^42^, macrophages^43^, cardiomyocytes^44^, lung epithelial cells^34^ and meningeal cells and astrocytes^45^. Rapid LDH release from cells infected with wt UPSA 807, but not its hemolysin-deficient mutant, suggests cytolytic non-apoptotic processes, such as pyroptosis, which can involve caspase-1 activation^46^,^47^. However, we did not detect significant caspase-1 activation in infected urothelial cells implicating non-pyroptotic cell death. While *S. agalactiae* can induce apoptosis in macrophages^42^,^48^,^49^ this pathogen is also cytotoxic towards respiratory epithelial cells and elicits reactive oxygen species and apoptosis that encompasses caspase-3 and -9^50^. The low-level but statistically significant activation of caspase-3 in urothelial cells in response to infection with UPSA 807 is of interest because apoptosis in urothelial cells occurs following infection with uropathogenic *E. coli*; the bacterial α-hemolysin activates caspase-3 in this cell death mechanism^26^,^51^. Together, the results of the current study show that the primary mechanism of host cell death induced by UPSA 807 in bladder cells is not via caspase activation, but instead, involves extensive cytolysis leading to rapid LDH release due to the expression of the bacterial β-H/C.

Interestingly, *S. agalactiae* β-H/C can also exert immunomodulatory effects on host cells including macrophages at sub-lytic concentrations *in vitro*^43^. Thus, the β-H/C can modulate innate immune functions beyond direct mechanisms of cell death. A recent study reported that *S. agalactiae* β-H/C promotes pro-inflammatory cytokine responses in bladder urothelial cells^19^ and our findings are consistent with this report. It would be of interest to examine the role of the nucleotide oligomerization domain (Nod)-like receptor (NLR) family, pyrin domain-containing 3 (NLRP3) inflammasome in this process since the β-H/C can promote the activation of the NLRP3 inflammasome and the production of IL-1β^42^. Another study showed that respiratory metabolism in *S. agalactiae* leads to higher cell numbers, driven by externally acquired haem and menaquinone^52^. This may be used to suggest that loss of β-H/C in UPSA 807 and attenuated host-cell lysis may compromise the bioavailability of host-derived haem *in vivo*, and affect the rates of respiration and cell division. Future studies would need to investigate this hypothesis.

The massive neutrophil infiltrate observed in the bladder in response to infection with wt UPSA 807, but not its hemolysin-deficient mutant, establishes that the β-H/C elicits an acute local cell infiltrate. These findings are supported by the chemokine responses in the bladder involving IL-6, G-CSF, KC, Rantes and MIP-1α, and are consistent with a study that showed neutrophil migration across brain microvascular endothelial cell monolayers triggered by *S. agalactiae* β-H/C in a process involving IL-8^33^. Neutrophils are important in IL-1 family cytokine production and processing^53^ and IL-1β aids in the control of *S. agalactiae* dissemination to target organs, including the kidneys^54^; this was associated with impaired KC, MIP-2α and neutrophil responses^54^.

The findings related to neutrophil infiltrates and bacterial survival in the bladder in the current study may be interpreted as a paradox given that major cellular infiltrates were associated with high bacterial loads for wt UPSA 807; in contrast, significantly lower bacterial loads for the β-H/C mutant were detected in parallel with a minimal neutrophil infiltrate. These data establish that while the β-H/C elicits cell infiltrates and contributes to the survival of UPSA 807 in the bladder *in vivo*, a massive neutrophil infiltrate does not necessarily enable efficient bacterial clearance. In other words, these data support the notion of subversion of innate immune defense mechanisms by *S. agalactiae*^13^, whereby the β-H/C mediates improved bacterial survival despite a massive neutrophil response that it elicits. The findings of a role for the β-H/C in bacterial survival contrast with a reported dispensable role for the β-H/C in the establishment of UTI in mice^19^. It is possible that differences in strains or experimental methods between the current and prior study^19^ may account for these contradictory findings. We demonstrated striking differences in the hemolytic activities of the UPSA and ABSA strains used in this study, however the level of hemolytic activity of the *S. agalactiae* strain used in^19^ is not reported. In addition, the previous study does not report a significant difference between wt and *cylE*^−^ strains in terms of cytotoxicity towards epithelial cells, perhaps signifying low-level hemolytic activity in this strain. Nonetheless, the findings of this study support a role for β-H/C in urothelial cell pro-inflammatory cytokine responses, as reported in^19^ and other virulence functions such as resistance to neutrophils^55^, and cytotoxicity towards epithelial cell barriers, which can lead to systemic infection^56^.

The findings of the current study are particularly interesting in the context of the clinical origin of the UPSA and ABSA strains. On the one hand, the phenotypes of adhesion, invasion, and cytotoxicity correlate with the clinical presentations of acute UTI versus ABU; i.e. UPSA 807 has greater adhesive and cytotoxic properties compared to ABSA 834 and ABSA 1014 both *in vitro* and *in vivo*. UPSA 807 also induces a stronger, more rapid cytokine response early following infection, particularly for IL-8/KC, Rantes and IL-6 (and IL-1β in human urothelial cells and MDMs but not mouse bladder) compared to ABSA 834. On other hand, both ABSA 834 and ABSA 1014 induced high levels of these cytokines, as well as multiple additional cytokines and chemokines, at 24 h p.i., including G-CSF, MIP-1α, TNF-α, IL-10, and IL-1β; these findings were unexpected and underscore the work that is needed to understand the basis of *S. agalactiae* ABU both clinically, and in experimental models. The degree to which these responses play a role in disease versus host defense is an area for future analysis. At the bacterial cell level, UPSA 807 is more hemolytic than ABSA 834 but less hemolytic than ABSA 1014. Notably, the finding that UPSA 807 is more cytotoxic towards urothelial cells compared to ABSA 1014 raises the intriguing question of the inability of ABSA 1014 to kill host cells despite high hemolytic activity. These data may reflect a different underlying host-pathogen interaction at the cell-cell interface, unique factors expressed in ABSA 1014 that promote host cell survival, or some intracellular responses that support host cell survival despite a high level of hemolytic activity. ABSA 1014 was cultured from an asymptomatic patient with ABU, which suggests that (i) hemolytic activity *in vitro* is not a useful surrogate marker of the acute UTI-causing potential of *S. agalactiae* strains, and (ii) hemolytic activity does not simply correlate with the degree of cytotoxicity towards eukaryotic cells. It is also possible that asymptomatic infection in the individual from whom ABSA 1014 was isolated was related to a reduced host response in the infected patient. There are some *S. agalactiae* factors other than the β-H/C that are also known to contribute to cytotoxicity; for example, lipoteichoic acid promotes internalization of bacteria and cytotoxicity in cardiomyocytes^57^. Finally, the complete absence of cytotoxicity observed in host cells infected with an isogenic β-H/C-deficient mutant of UPSA 807, in contrast to infection with the wt strain, shows a predominant role for this factor in the death of urothelial cells. This finding, that UPSA-driven cytotoxicity in urothelial cells is mediated almost entirely by the β-H/C, suggests a key role for this toxin in acute UTI at the interface between bacteria and the bladder.

In summary, we conclude that *S. agalactiae* UTI pathogenesis is subject to bacterial strain influences that affect adhesion to, invasion and killing of bladder cells, and the β-H/C that promotes urovirulence by eliciting inflammation and bacterial survival in the bladder. UPSA 807 is more adherent, invasive and cytotoxic towards urothelial cells compared to ABSA 834 and ABSA 1014. The mechanisms of host cell death mediated by UPSA 807 include low-level caspase-3 activation, and cytolysis. UPSA 807-induced cytotoxicity is mediated by the β-H/C. Collectively, the findings of this study support a model of *S. agalactiae* UTI in which the β-H/C is a key factor in facilitating disease pathogenesis, bacterial virulence, and evasion of local innate defense in the bladder.

## Methods

### Bacterial strains, plasmids and primers

The previously characterized, clinically isolated strains of UPSA 807, ABSA 834 and ABSA 1014 used in this study are described elsewhere^22^. Uropathogenic *S. agalactiae* (UPSA) strain 807 was cultured from clean catch voided urine from a 59-year-old woman who presented with frequency, urgency, haematuria, and pyuria. Urinalysis was consistent with a clinical diagnosis of acute uncomplicated cystitis. ABU-causing *S. agalactiae* (ABSA) strain 834 was cultured from urine from an asymptomatic 57-year-old woman undergoing routine screening. Repeat urine cultures grew *S. agalactiae* over a 15-month period. ABSA 1014 was cultured from urine obtained by catheter from an asymptomatic 26-year-old pregnant woman undergoing routine screening. Repeat urine cultures grew *S. agalactiae* over a 5-week period. Written informed consent was obtained from study participants according to approvals from the University of Alabama at Birmingham Institutional Review Board (approval X070722011) and the Griffith University Human Research Ethics Committee (approval MSC/02/11/HREC), as previously described^4^,^22^. The methods used in all studies involving human subjects were carried out in accordance with the approved guidelines of the University of Alabama at Birmingham, Griffith University and the Australian National Health and Medical Research Council (NHMRC). A β-H/C-deficient mutant of UPSA 807 (UPSA 807Δ*cylE*) was generated as described below. Additional details of bacterial strains, plasmids and primers are listed in Supplementary Table S1. Bacteria were routinely grown in Todd Hewitt broth (THB; Thermo Fisher Scientific, Scoresby, VIC, Australia) at 37 °C with shaking at 200 rpm, and on Todd Hewitt agar at 37 °C.

### *In vitro* assay

Human 5637 urothelial cells, used for adhesion and invasion assays, were grown and infected as previously described^15^, with minor modifications. The antibiotic concentrations required to kill extracellular *S. agalactiae* were determined by minimum inhibitory concentration assay as 250 U mL^−1^ for penicillin, 250 U mL^−1^ for streptomycin and 50 μg mL^−1^ for gentamicin, which were used in combination unless otherwise indicated. 5637 cells were seeded at a density of 3.0 × 10^5^ cells in wells of a 24-well tissue culture-treated plate (Nunc, Rochester, New York, USA), and grown for 24 h at 37 °C in 5% CO_2_. Two multiplicity of infection (MOI) ratios were used; 50 or 200 bacteria per cell. Culture supernatants were collected and analyzed for lactate dehydrogenase (LDH) using the TOX7 toxicology assay (Sigma-Aldrich, Castle Hill, NSW, Australia), as a measure of cell lysis, or used for cytokines measurements (IL-1β, IFN-γ, IL-10, IL-8, IL-6, IL-17A and TNF-α) using the Ready-SET Go! ELISA Kit (eBioscience, San Diego, USA). Cells stained with trypan blue were used to enumerate viable and non-viable cells. In parallel, we performed infection assays using human monocyte-derived macrophages (MDMs) matured from U937 monocytes using phorbol-12-myristate-13-acetate (PMA) for 24 h (10 μM) and rested 5 days in culture without PMA, as previously described^58^. Monocytes were seeded for maturation at a density of 5.0 × 10^5^ cells per well and, following the rest period, were infected as for epithelial cells. In co-culture experiments, urothelial cells and MDMs were mixed in the proportion 1:10 using 4.8 × 10^5^ urothelial cells and 2.0 × 10^4^ MDMs. The adhesion assay was performed by infecting monolayers in antibiotic-free media for 1 h, followed by extensive washing of the infected monolayers (5 times with PBS). The monolayers were then lysed by the addition of 0.1% Triton-X 100 and used for colony counts on agar. Colony counts of cell lysates collected at 3 h p.i. were performed to measure invasion. Intracellular persistence was determined by enumerating the bacteria surviving at 24 h p.i. in culture maintained in media supplemented with antibiotics, as above.

### Caspase-activity assay

The mechanism of infection-induced cell death was studied by staining urothelial cells for active caspase-3 and measuring the enzymatic activities of the upstream caspases, -1, -8, and -9. For immunohistochemistry staining of active caspase-3, the cells were prepared on coverslip inserts in 24-well culture plates and, at 5 h p.i. (infection as above), were stained for active caspase-3 (rabbit pAb G7481, 1/250; Promega, Alexandria NSW, Australia), anti-rabbit IgG secondary Ab Alexa Fluor 488 conjugate (A-11008, 1/100; Life Technologies, Mulgrave, VIC, Australia), and Hoechst 33258 (20 μg mL^−1^ in PBS; Sigma-Aldrich, Castle Hill, NSW, Australia) for co-staining of nuclei. Cells treated with 10 μM staurosporine for 5 h were used as a positive control. Samples were mounted in 0.2% n-propyl gallate. Images were acquired using an AxioImager.M2 microscope (Carl Zeiss MicroImaging, Oberkochen, Germany) fitted with Plan-Apochromat ×63/1.40, Plan-Apochromat 20×/0.8 and Plan-Neofluar 10×/0.3 objectives, a AxioCam MRm Rev.3 camera, and Zen 2012 SP2 Imaging Software. For tiling of numerous fields, twelve or more fields of view were captured at 20X magnification using a motorized scanning stage fitted to the microscope; the images were stitched together using the tiles/positions module in Zen software. For additional caspase activity assays, supernatants prepared from cell lysates collected from infected and non-infected urothelial cells at 6 h p.i., were analyzed for caspase-1, 3, 8 and 9 activity using Fluorometric Caspase Activity Kits (Abcam, Melbourne, VIC, Australia).

### Murine model of UTI

A murine model of UTI, utilizing female C57BL/6 mice (8–10 weeks; Animal Resources Centre [Canning Vale, WA, Australia]), and the standard method of transurethral infection^59^ was used with minor modifications, essentially as described elsewhere^16^. Mice were infected with 5 × 10^8^ CFU of UPSA 807, ABSA 834 or ABSA 1014 in 40–50 μl PBS. Urine samples and bladders were collected at 6 h and 24 h p.i. and processed for colony counts or used for 23-target multiplex protein assay (Bio-Rad, Gladesville, NSW, Australia). The methods used in all animal studies were carried out in accordance with the approved guidelines of Griffith University and the Australian NHMRC. All experimental protocols for animal experiments were approved by Griffith University Animal Ethics Committee (approval: MSC/03/12/AEC). For multiplex protein analysis, the bladders were homogenized in PBS containing protease inhibitor (Complete Ultra Tablet mini, EDTA free, Roche, Melbourne, VIC, Australia). For histology, the bladders were fixed in 10% neutral buffered formalin, embedded in paraffin, cut in 5 μm-thick sections, and stained with hematoxylin and eosin (H&E) according to standard techniques. Alternatively, sections were stained for neutrophils using a primary Gr-1 antibody (rat anti-mouse Ly-6G clone NMP-R14, 1/120; Abcam, Melbourne, VIC, Australia) overnight at room temperature, followed by Rat Probe (neat) and Rat-on-Mouse HRP-Polymer (Biocare Medical, Concord, California, USA) and developed with betazoid DAB. Sections were counterstained in Haematoxylin, washed in water, dehydrated through ascending graded alcohols, cleared in xylene, and mounted using Depex (Southern Biological, Knoxfield, VIC Australia). Images were acquired on a AxioImager.M2 microscope.

### Generation of β-H/C-deficient UPSA 807 mutant

β-H/C-deficient UPSA 807 was generated by replacement of the β-H/C *cylE* gene with a chloramphenicol cassette. We used pHY304-aad9 for homologous recombination, as previously described^22^,^48^. 886 bp  and 915 bp fragments corresponding to the sequence 5′ and 3′ of the *cylE* coding sequence, respectively, were amplified by PCR using the primer pairs (obtained from Sigma-Aldrich, Castle Hill, NSW, Australia) listed in Table S1. A 871 bp chloramphenicol resistance cassette of pLZ12 was amplified using primers CmAmpliF1 and CmAmpliR1^22^, and used for overlapping PCR using primers cylE-Up-F1 and cylE-Down-R1 to generate a 2.65 kb product. This was digested with *PstI* and ligated using T4 DNA Ligase (New England Biolabs, Arundel, QLD, Australia) into pHY304-aad9 to form pGU2351, which was electroporated into UPSA 807. Transformants, selected on agar at 30 °C with spectinomycin (100 μg mL^−1^), were grown at 37 °C without antibiotics, and single colonies were isolated by selection with chloramphenicol (10 μg mL^−1^) at 37 °C and screened for the loss of spectinomycin resistance. Colonies that were sensitive to spectinomycin were screened for mutation in *cylE* by PCR using primers cylE-Chk-F1 and cylE-Chk-R1. PCR products were sequenced in their entirety to confirm allelic replacement of 1680 bp of *cylE*. A chloramphenicol resistant β-H/C-deficient isolate of UPSA 807 (UPSA 807Δ*cylE*) was termed GU2417.

### Hemolytic activity assays

Assays were performed essentially as described elsewhere^60^, with minor modifications. Briefly, 10 ml overnight THY cultures were washed twice in PBS and resuspended in 10 ml of PBS + 0.2% glucose. One hundred microliter (approx. 10^7^ CFU) aliquots, dispensed into wells of a 96-well plate, were mixed with an equal volume of 1% (vol/vol) horse erythrocytes (Thermo Fisher Scientific, Scoresby, VIC, Australia), suspended in PBS-glucose, and incubated at 37 °C for 5 h. As positive and negative controls, 100 μl 2% Triton-X 100 or PBS-glucose were used, respectively. At each time point, clarified supernatants were diluted 1:5 in PBS and hemoglobin release was measured at OD420 nm. Data are shown as erythrocyte lysis as a % of the positive control.

### Statistical analysis

*In vitro* adhesion, invasion, and cytotoxicity data, as well as the levels of cytokines detected in *in vitro* assays, were compared using Kruskal-Wallis ANOVA tests for multiple comparisons, independent samples student’s t-tests for pair-wise comparisons, or Mann Whitney U tests for pair-wise comparisons of specific data that exhibited unequal distributions and non-homogeneity of variance. Time-course profiles for *in vitro* data were also compared using area-under-curve (AUC) analysis with student’s t-tests. *In vivo* colonization data were compared using a Mann Whitney U test (data that exhibited unequal distributions and non-homogeneity of variance) and independent samples student’s t-tests for pair-wise comparisons (data that exhibited equal distributions and homogeneity of variance). Group wise data are displayed with mean ± SEM or median ± interquartile range according to the analysis for each dataset. The statistical analyses were performed using GraphPad Prism v6 and SPSS v21.0.

## Additional Information

**How to cite this article**: Leclercq, S. Y. *et al*. Pathogenesis of *Streptococcus* urinary tract infection depends on bacterial strain and β-hemolysin/cytolysin that mediates cytotoxicity, cytokine synthesis, inflammation and virulence. *Sci. Rep*. **6**, 29000; doi: 10.1038/srep29000 (2016).

## Supplementary Material

Supplementary Information
