# Supplementary material for: Pathogenesis of Streptococcus urinary tract infection depends on bacterial strain and β-hemolysin/cytolysin that mediates cytotoxicity, cytokine synthesis, inflammation and virulence
Source: Sci Rep. 2016 Jul 7;6:29000. doi: 10.1038/srep29000 (PMC4935997; doi:10.1038/srep29000)
Supplement: Supplementary Information [file srep29000-s1.pdf]

Supplementary Information

Pathogenesis of *Streptococcus* urinary tract infection depends on bacterial strain and β-hemolysin/cytolysin that mediates cytotoxicity, cytokine synthesis, inflammation and virulence

Sophie Y. Leclercq[a,b], Matthew J. Sullivan[a], Deepak S. Ipe[a,], Joshua P. Smith[a] Allan W. Cripps[a], and Glen C. Ulett[a #]

[a]School of Medical Science, and Menzies Health Institute Queensland, Griffith University, Parklands, Australia 4222; [b]Research and Development Center, Ezequiel Dias Foundation (Funed), Belo Horizonte, MG, Brazil.

Supplementary Table S1. Strains, plasmids, and primers.

Strain/Plasmid /Primer	Features/Sequence [b]	Reference/ Usage
**Strain**		
UPSA 807	Uropathogenic *S. agalactiae*, type V; 1 (1)[a], resistant to gentamicin, clindamycin, tetracycline	Ipe et al [22]
ABSA 834	ABU-causing *S. agalactiae*, type III; 182 (19)[a], resistant to gentamicin	Ipe et al [22]
ABSA 1014	ABU-causing *S. agalactiae*, type II; 28 (19)[a], resistant to gentamicin	Ipe et al [22]
GU2351	*E. coli* K-12 carrying pGU2351 targeting vector	This work
GU2417	UPSA 807-derivative, *cylE*$^-$ mutant, Cm^r	This work
DH5α	*E. coli* K-12 strain, *fhuA2 Δ(argF-lacZ)U169 phoA glnV44 f80Δ(lacZ)*M15 *gyrA96 recA1 relA1 endA1 thi-1 hsdR17*	
**Plasmid**		
pHY304*aad9*	*ori*(TS), Temperature-sensitive vector, Spt^r	Ipe et al [22]
pLZ12	Streptococcus shuttle vector, Cm^r	
pGU2351	pHY304*aad9*-derivative, *cylE*-mutation construct, Spt^r Cm^r	This work
**Primer**		
CylE-Up-F1	**GATC*CTGCAG***GATCGGTTAGCGTTTTTCATG (*Pst*I)	pGU2351
CylE-Up-R1	CCAATTTTCGTTTGTTGAACTAAAGAATAATCTTCTAAGGAAGC	pGU2351
CylE-Down-F1	CTAATGTCACTAACCTGCCCCTTTGATTATCGAATTAGAGG	pGU2351
CylE-Down-R1	**GATC*CTGCAG***GTGAAATCTAGATTCCAAG (*Pst*I)	pGU2351
cylE-Chk-F2	TAGAGGCGGCGTATCTCAAC	sequencing
cylE-Chk-R2	AGGGCTTGATTGTGTTTTCAA	sequencing
CmR-Up-R1	CGTTTGTTGGTTCAAATAATGA	sequencing
CmR-Dn-F1	TTCCATGGACTTCATTTACTGG	sequencing

[a] type lists serotype; sequence type (clonal complex)

[b] underlined bases are homologous to Cm^r-cassette of pLZ12, bold text denote bases not found in GBS and italicised bases indicate restriction enzyme sites.

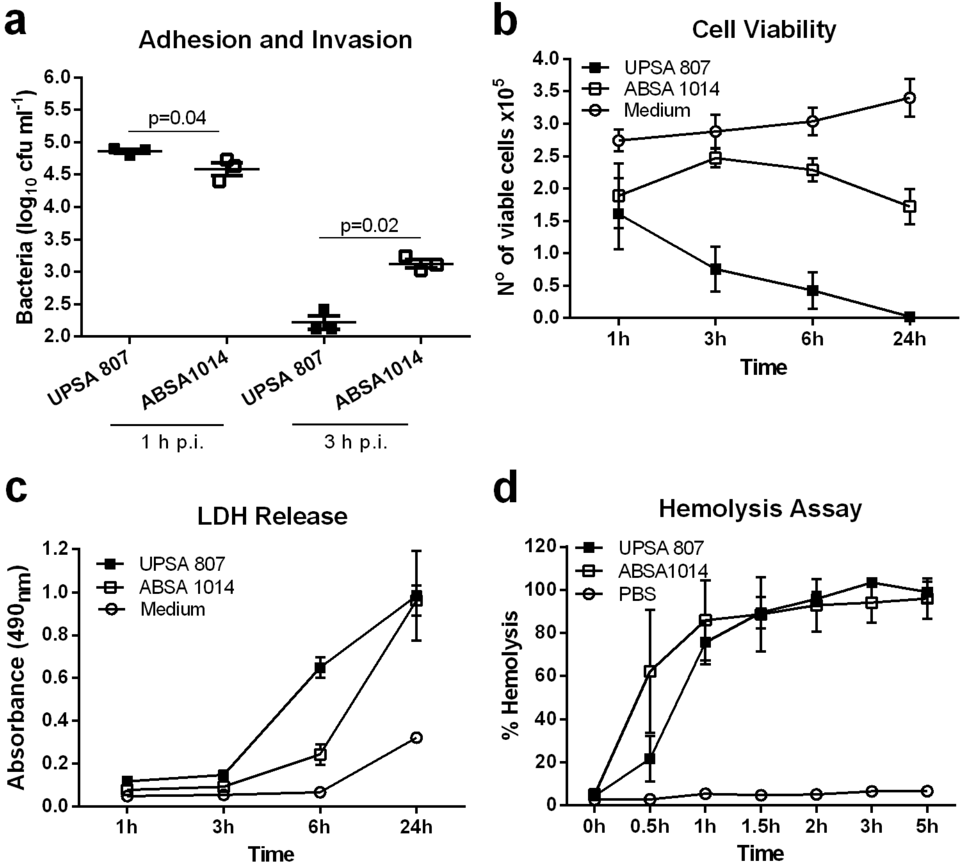
a
Adhesion and Invasion
Bacteria (log10 cfu ml-1)
6.0
5.5
5.0
4.5
4.0
3.5
3.0
2.5
2.0
p=0.04
p=0.02
UPSA 807
ABSA1014
UPSA 807
ABSA1014
1 h p.i.
3 h p.i.
b
Cell Viability
N° of viable cells x10^5
4.0
3.5
3.0
2.5
2.0
1.5
1.0
0.5
0.0
UPSA 807
ABSA 1014
Medium
1h
3h
6h
24h
Time
c
LDH Release
Absorbance (490nm)
1.2
1.0
0.8
0.6
0.4
0.2
0.0
UPSA 807
ABSA 1014
Medium
1h
3h
6h
24h
Time
d
Hemolysis Assay
% Hemolysis
120
100
80
60
40
20
0
UPSA 807
ABSA1014
PBS
0h
0.5h
1h
1.5h
2h
3h
5h
Time

Supplementary Figure S1. Adhesion, invasion, cytotoxicity and hemolytic activity phenotypes of ABSA 1014 compared to UPSA 807. Adhesion and invasion measures were assessed at 1 h and 3 h p.i., respectively using urothelial cells at a MOI of 50 (a). Cytotoxicity was measured as cell viability (b) and LDH release (c) that were quantified from urothelial cells challenged with ABSA 1014, UPSA 807, or RPMI medium. Data are pooled from three to four independent experiments each containing at least quadruplicate samples, and compared using independent samples t-test (a) or AUC students t-test (b-c) with P-values displayed.

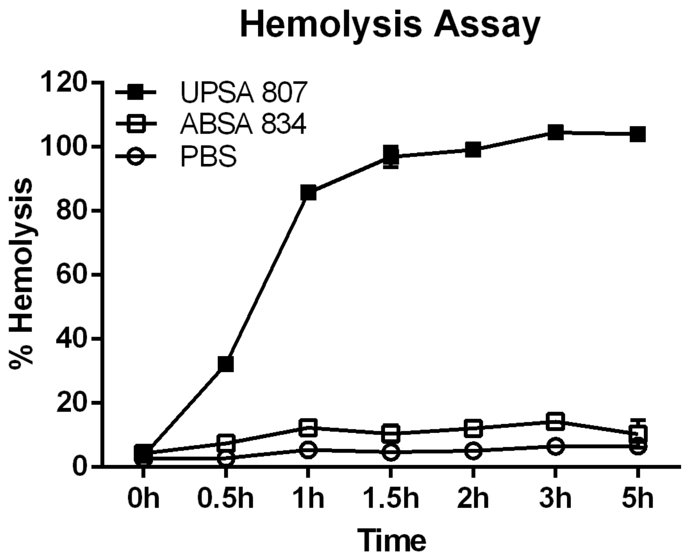
Hemolysis Assay
% Hemolysis
120
100
80
60
40
20
0
UPSA 807
ABSA 834
PBS
0h
0.5h
1h
1.5h
2h
3h
5h
Time

Supplementary Figure S2. The hemolytic activities of UPSA 807 compared to ABSA 834. Erythrocytes exposed to 2% Triton-X 100 were used as a positive control. Data are shown from one independent experiment, representative of two, each containing duplicate samples.

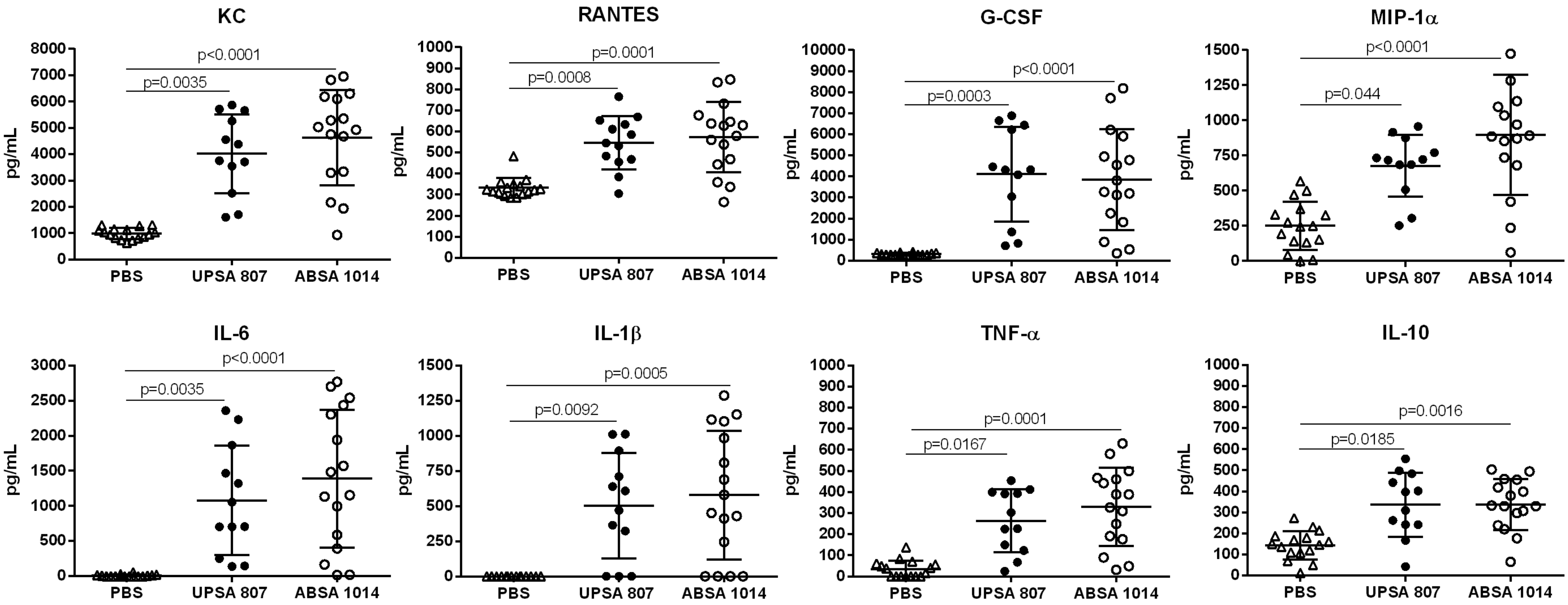
KC
p<0.0001
p=0.0035
pg/mL
PBS
UPSA 807
ABSA 1014
RANTES
p=0.0001
p=0.0008
G-CSF
p<0.0001
p=0.0003
MIP-1α
p<0.0001
p=0.044
IL-6
p<0.0001
p=0.0035
IL-1β
p=0.0005
p=0.0092
TNF-α
p=0.0001
p=0.0167
IL-10
p=0.0016
p=0.0185

Supplementary Figure S3. Cytokine production in the murine model of UTI with ABSA 1014 and UPSA 807. A selection of cytokines were quantified in bladder homogenates that were prepared from mice infected with UPSA 807 and ABSA 1014 and compared to control mice (PBS) at 24 h p.i. Data are pooled from two independent experiments (each containing 6 mice) and compared using Kruskal-Wallis ANOVA with Tukey's multiple comparison test with P-values displayed.
